# Predictive Value of C-Reactive Protein/Albumin Ratio in Acute Pancreatitis

**DOI:** 10.7759/cureus.93436

**Published:** 2025-09-28

**Authors:** Nischal Khanal, Amrit Baral, Shristi Shrestha, Paleswan Joshi Lakhey, Ramesh Singh Bhandari

**Affiliations:** 1 General Surgery, James Paget University Hospitals NHS Foundation Trust, Lowestoft, GBR; 2 Public Health Sciences, Miller School of Medicine, University of Miami, Miami, USA; 3 Intensive Care Unit, Madan Bhandari Academy of Health Science, Hetauda, NPL; 4 Surgical Gastroenterology, Maharajgunj Medical Campus, Institute of Medicine, Tribhuvan University Teaching Hospital, Kathmandu, NPL; 5 Surgery, Maharajgunj Medical Campus, Institute of Medicine, Tribhuvan University Teaching Hospital, Kathmandu, NPL

**Keywords:** acute pancreatitis, albumin, c-reactive protein, revised atlanta classification, severity

## Abstract

Background

Acute pancreatitis (AP) is a common and potentially life-threatening condition with a wide spectrum of severity. While most cases are mild, a subset progresses to severe disease with high mortality. This study evaluates the predictive value of the C-reactive protein (CRP)/albumin ratio (CAR), an inflammation-based biomarker, in determining AP severity.

Methods

A prospective, observational study was conducted among adult patients diagnosed with AP. CRP and albumin levels were measured at presentation, and CAR was calculated and categorized into three quantiles (≤6.7, 6.7-30.5, ≥30.5). Associations between CAR quantiles and disease severity were analyzed using Fisher’s exact test. Pearson correlation assessed the relationship between CAR and hospital stay. Receiver operating characteristic (ROC) analysis was performed to evaluate diagnostic performance, with optimal cutoffs determined by Youden’s index. Sensitivity, specificity, positive predictive value (PPV), and negative predictive value (NPV) were calculated at both the Youden cutoff and a lower pragmatic threshold.

Results

Among the 80 participants (55% male; median age 44.5 years), the median CAR was 12.7. No patients in the lowest CAR quantile developed severe AP. CAR demonstrated good discriminatory ability for severe AP (AUC 0.862). The optimal Youden cutoff of 18.39 yielded 100% sensitivity and 69.4% specificity. At a lower cutoff of 10.28, sensitivity remained 100%, while specificity decreased to 40.3%. CAR values were positively correlated with hospital stay duration (r = 0.568, p < 0.001).

Conclusion

CAR is a simple, inexpensive, and non-invasive marker that predicts AP severity. Low CAR values are associated with a reduced risk of severe AP. Using CAR, particularly at a lower threshold, may help identify high-risk patients early and guide management decisions in resource-limited settings.

## Introduction

AP continues to be a common and potentially fatal disease, with numerous causes, obscure pathogenesis, nonspecific treatment, and often unpredictable prognosis [1]. AP is a common inflammatory condition in Nepal [2,3], with alcohol and gallstones being the primary etiologies [2-4]. It predominantly affects males aged 40-60 years [2].

AP is an inflammatory condition of varied etiology. Yet, each produces a similar disease pattern, indicating that they all converge at a common point to initiate a cascade of events resulting in AP [5]. Moreover, AP has a broad spectrum of clinical presentations, ranging from mild discomfort to apocalyptic prostration. Most episodes of AP are mild and self-limiting and do not require any intervention [6]. However, 15-25% of all patients with acute pancreatitis develop severe AP, and mortality rates remain much higher in subgroups of patients with severe disease, exceeding 30% [7-8]. The ability to predict the severity of AP can help identify patients at increased risk for morbidity and mortality, thereby assisting in the appropriate early triage of patients to intensive care units and selecting patients for specific interventions [9]. For assessing AP severity, the “Atlanta Classification” has been considered the global standard tool since its creation in 1992 [10]. However, over time, some of the original Atlanta Classification's definitions have been ambiguous, especially its definition of “severity.” In 2012, the Atlanta classification was revised with an emphasis on persistent organ failure [11].

Multi-factorial scoring systems, including those developed by Ranson et al. and the Acute Physiology and Chronic Health Evaluation (APACHE)-II, have been used since the 1970s to assess the severity of acute pancreatitis (AP) [12]. These predictive methods have been established as a crucial tool to evaluate the severity of AP [13]. However, these multi-factorial scoring systems, which are complex and difficult to use clinically, have been shown to perform with a high negative predictive value but only moderate overall sensitivity [14]. For this reason, there is a need for easy-to-use and inexpensive indices that can determine disease severity and indicate prognosis within minutes in clinical practice.

C-reactive protein (CRP) is a positive acute-phase reactant synthesized by the liver, and its level in the blood rises in response to inflammation and infection within hours [11]. It is frequently assessed as a biomarker in follow-ups of medical conditions with infection and inflammation due to its short half-life, easy measurement, and close relationship with the prognosis of the disease [15]. It can be used for diagnosis, treatment follow-up, and mortality prediction, particularly in cases involving inflammation [16]. Conversely, albumin is a negative acute-phase reactant produced by the liver, and its blood level drops during inflammation [15]. The CRP/albumin ratio (CAR) is a novel, inflammation-based prognostic score that is linked to inflammation severity and mortality [17]. The present study investigated the prognostic value of the CAR in predicting the severity of acute pancreatitis.

## Materials and methods

Data source and study sample

This hospital-based, prospective, observational study was conducted at the Department of Gastroenterology (GI) and General Surgery, Tribhuvan University Teaching Hospital (TUTH), Kathmandu, Nepal, between September 2018 and August 2019. It was approved by the Institutional Review Committee, Tribhuvan University Institute of Medicine (approval number: 245(6-11-E)2/075/076).

Eligible participants were individuals aged 18 years or older who presented to the Emergency Room or Outpatient Department, were diagnosed with acute pancreatitis (AP), and provided informed consent. Among 88 patients initially recruited, 80 were included in the final analysis. the remaining eight patients were excluded due to pregnancy (n = 2), age <18 years (n = 1), urinary tract infection (n = 2), sickle cell disease (n = 1), diabetic ketoacidosis (n = 1), and hepatitis C (n = 1). The final sample size of 80 was determined based on a calculation using a 5% [18] prevalence of acute pancreatitis, a 95% confidence interval (CI), and a margin of error of 0.05.

The diagnosis of AP was made by surgical residents based on characteristic abdominal pain and elevation in serum amylase levels ≥3 times the upper normal limit, in accordance with established diagnostic criteria and the revised Atlanta classification [11]. Blood samples for CRP, albumin, and other investigations were collected at admission. The CAR was calculated accordingly.

Definitions

The primary outcome was the severity of acute pancreatitis (mild, moderate, or severe), classified according to the modified Atlanta classification [11]. For diagnostic performance analysis, severity was dichotomized into severe versus non-severe. The secondary outcome was the length of hospital stay (in days). The primary exposure variable was CAR, which was categorized into quantiles: ≤6.73, 6.73-30.5, and ≥30.5. Covariates included age, sex, and etiological factors for AP.

Statistical analysis

Descriptive statistics were computed to summarize baseline characteristics. Differences across CAR quantiles were evaluated using Chi-squared or Fisher’s exact tests for categorical variables, and Mood’s median test for continuous variables. The relationship between CAR quantiles and AP severity was assessed using Fisher’s exact test. The correlation between CAR and hospital stay duration was analyzed using Pearson correlation.

To evaluate the diagnostic utility of CAR for identifying severe AP, receiver operating characteristic (ROC) curve analysis was performed, and the area under the curve (AUC) was calculated. Since complete severity classification was available for 76 out of 80 patients, only these 76 patients were included in the ROC analysis. Optimal cutoff values were determined using Youden’s index. Sensitivity, specificity, positive predictive value (PPV), and negative predictive value (NPV) were calculated at both the Youden cutoff and a lower cutoff of 10.28, selected as a potentially pragmatic threshold for early detection.

All tests were two-tailed, and statistical significance was set at p < 0.05. Analyses were conducted using SAS software, version 9.4 (SAS Institute Inc., Cary, NC, USA). All participants provided informed consent, and data were anonymized prior to analysis.

## Results

Sample characteristics

The study sample consisted of 80 participants with a median age of 44.5 years (range: 18-80). There was no significant difference in median age between study participants by quantiles (p=0.12) (Table 1).

**Table 1:** Age distribution of the study sample by quantiles of CAR values (N=80) *p-value was obtained using Mood’s Median Test CAR: C-reactive protein/albumin ratio

Age (years)	Median (range)	Quantile of CAR values			p-value*
		Lower 25% (n=20)	Middle 50% (n=40)	Upper 25% (n=20)	
	44.5 (18-80)	38.5	51.5	49.0	0.12

The majority of the participants were male (55%). Sex distribution across the CAR quantiles revealed significant differences (p<0.01). In the lower 25% CAR quantile, the male-to-female ratio was approximately equal, with 55.0% males and 45.0% females. However, in the middle 50% CAR quantile, there was a higher proportion of females (60.0%) than males (40.0%). This pattern was reversed in the upper 25% CAR quantile, where males constituted 85.0% (Table 2).

**Table 2:** Sex distribution of the study sample by quantiles of CAR value (N=80) ^a^p-value was obtained using Chi-squared Test CAR: C-reactive protein/albumin ratio

Sex	Frequency (Percentage)	Quantiles of CAR values			Chi-Squared (X^2^) value	p-value^a^
		Lower 25% (n=20)	Middle 50% (n=40)	Upper 25% (n=20)		
Male	44 (55.0)	11 (55.0)	16 (40.0)	17 (85.0)	10.91	0.0027
Female	36 (45.0)	9 (45.0)	24 (60.0)	3 (15.0)		

Clinical characteristics

**Figure 1:**
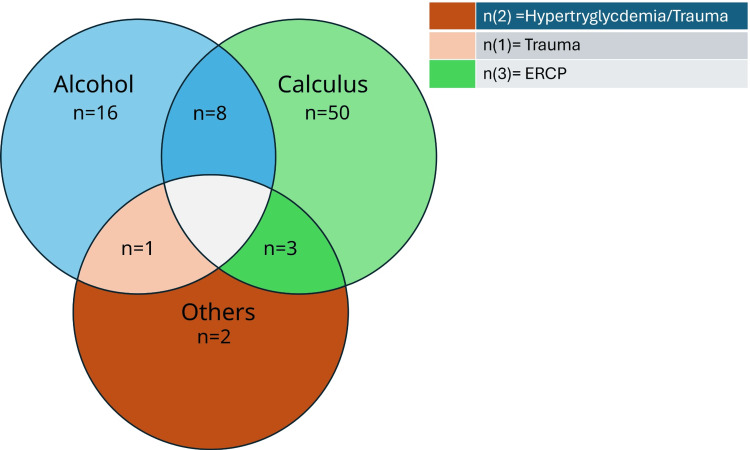
Etiologies of acute pancreatitis in the study participants (N=80)

The predominant etiology of AP in our study participants was gallbladder (GB) calculus (n=61). Alcohol consumption was the second most common cause (n=25). Notably, GB calculus was the primary cause in 58 patients. Additionally, eight patients with a history of daily alcohol consumption also had GB calculus, indicating a combined etiology (Figure 1).

Acute pancreatitis occurring as a complication during therapeutic endoscopic retrograde cholangiopancreatography (ERCP) for choledocholithiasis was observed in three patients. Other etiologies included trauma in two cases and hypertriglyceridemia in one case. Abdominal pain was universal, while vomiting occurred in 64 patients and fever in seven.

**Figure 2:**
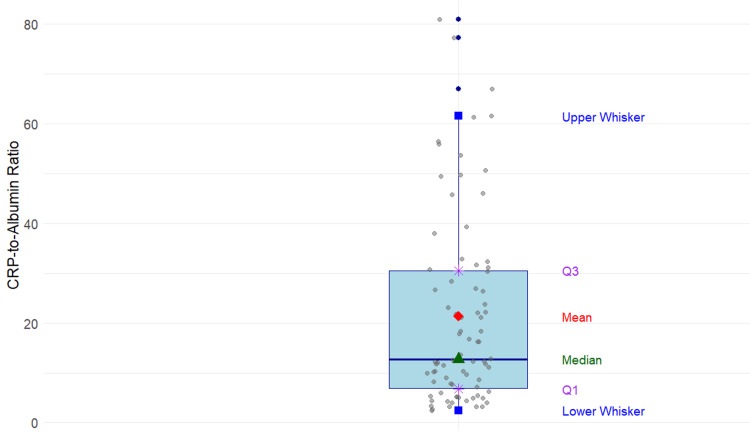
Box-and-whisker plot showing the distribution of CAR values (N=80) CAR: C-reactive protein/albumin ratio

According to the Revised Atlanta Classification, 48 cases were mild, 18 moderately severe, and 14 severe. Sixteen local complications were documented: pancreatic necrosis (n=9), walled-off necrosis (n=1), and acute peripancreatic fluid collection (n=6). The mean serum albumin value was 3.4 g/L (SD=0.50). The median CAR value was 12.7 mg/dl (range: 12.4-80.9). The mean presentation duration since the onset of symptoms was 3.6 days (SD = 3.9). Finally, the mean duration of hospitalization was 6.2 days (range: 0-28 days). A box-and-whisker plot was constructed to represent the distribution of CAR values, as shown in Figure 2.

**Figure 3:**
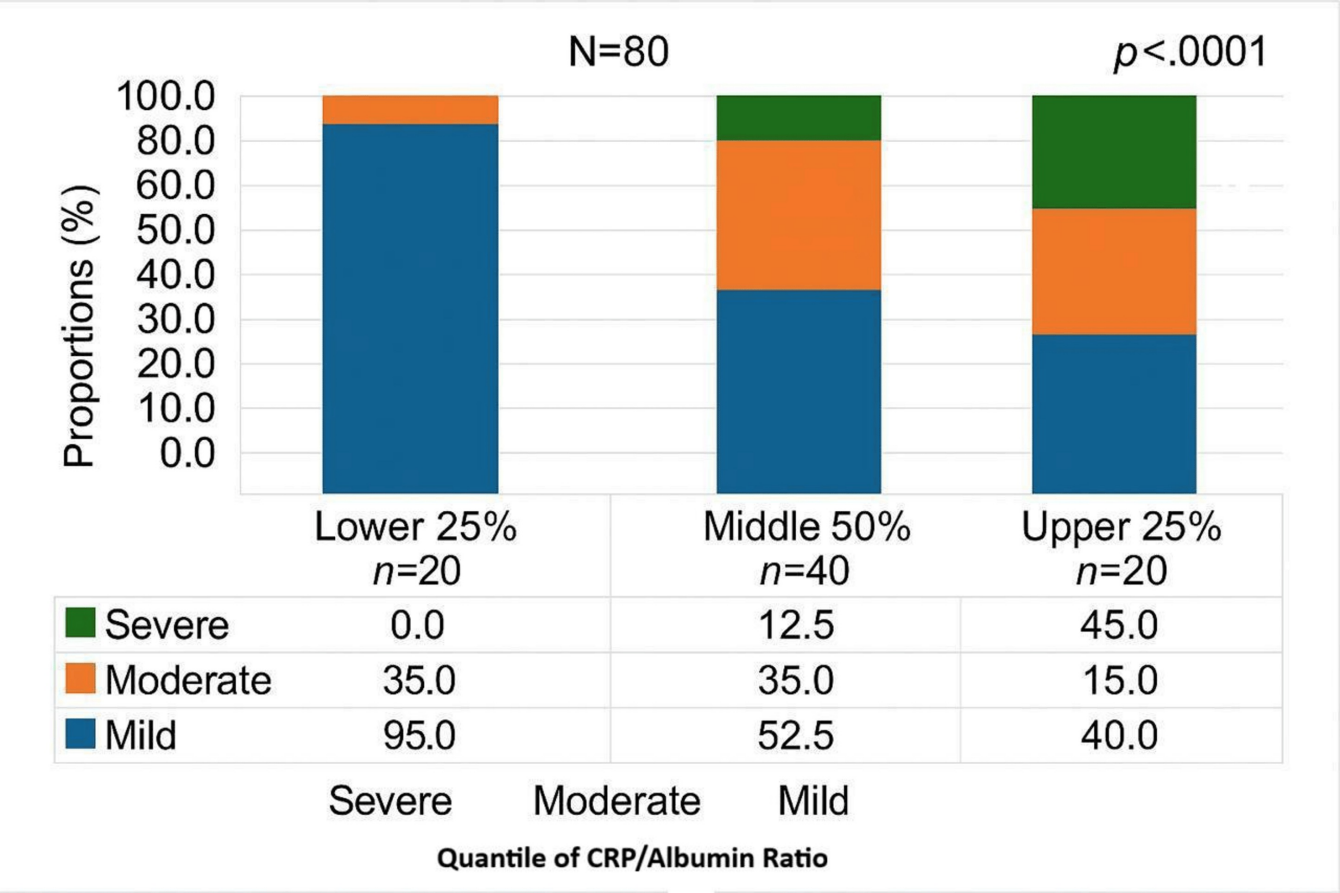
Stacked column chart showing the association between CAR quantiles and the severity of AP (N=76) p-value obtained from Fisher’s exact test CAR: C-reactive protein/albumin ratio; AP: acute pancreatitis

The CAR values ranged from 2.4 to 80.9. The first quartile (Q1) was 6.7, and the third quartile (Q3) was 30.5. The median CAR value was 12.7. The distribution of AP severity across CAR quantiles is shown in Figure 3.

**Figure 4:**
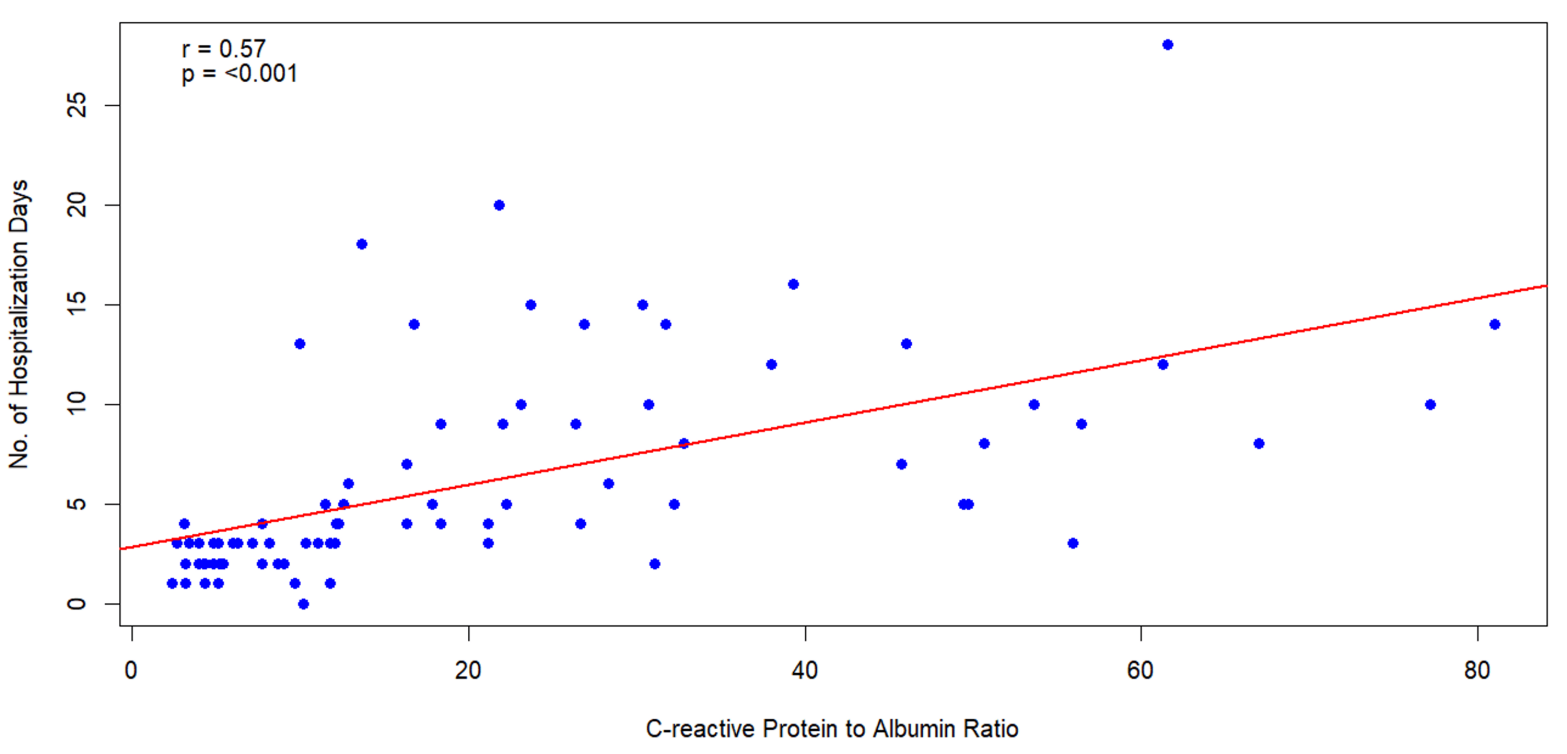
Scatter plot showing the correlation between the duration of hospital stay and the CAR value CAR: C-reactive protein/albumin ratio

In the lowest quantile of CAR values (≤6.7), 95% of patients had mild pancreatitis, and 5% had moderate pancreatitis; no cases of severe AP were observed in this group. In the middle quantile (CAR range 6.7-30.5), 52.5% of patients had mild pancreatitis, 35% had moderate pancreatitis, and 12.5% had severe pancreatitis. In the highest quantile (CAR >30.5), 40% of patients had mild pancreatitis, 15% had moderate pancreatitis, and 45% had severe pancreatitis. Fisher’s exact test confirmed a significant association between CAR quantiles and AP severity (p<0.0001). Additionally, Pearson’s correlation test demonstrated a statistically significant positive correlation between the duration of hospital stay and CAR values (r = 0.568, p < 0.001), as shown in Figure 4.

**Figure 5:**
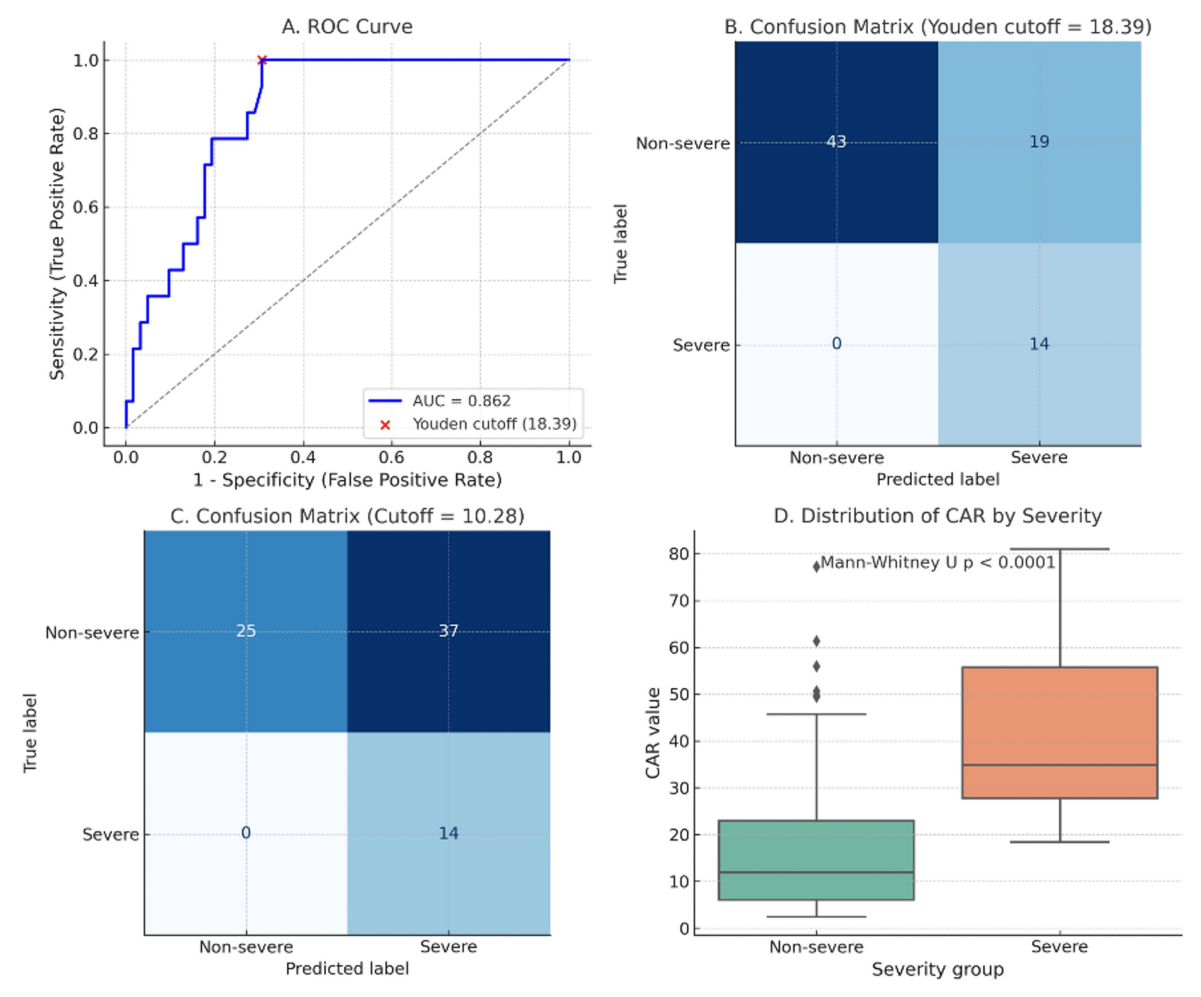
Diagnostic performance of the CAR for severe pancreatitis. (A) ROC curve showing discriminatory ability of CAR (AUC = 0.862) with the optimal cutoff (18.39) identified by Youden’s index. (B) Confusion matrix at the Youden cutoff (18.39), yielding 100% sensitivity and 69.4% specificity. (C) Confusion matrix at a lower cutoff (10.28), maintaining 100% sensitivity but reducing specificity to 40.3%. (D) Distribution of CAR values in severe versus non-severe cases, with significantly higher CAR values observed in severe pancreatitis (Mann–Whitney U test, p < 0.0001). CAR: C-reactive protein/albumin ratio; ROC: receiver operating characteristic; AUC: area under the curve

Of the 80 patients enrolled, severity classification was available for 76 patients. Accordingly, only these 76 patients were included in the ROC analysis. Among them, 14 (18.4%) had severe pancreatitis. CAR demonstrated good discriminatory capacity with an AUC of 0.862 (Figure 5).

The optimal cutoff identified by Youden’s index was 18.39, yielding 100% sensitivity and 69.4% specificity. At a lower cutoff of 10.28, sensitivity was maintained at 100%, but specificity declined to 40.3%, resulting in more non-severe cases being misclassified as severe. Importantly, at both thresholds, no severe cases were missed, underscoring CAR’s value as a highly sensitive marker for identifying patients at risk of severe disease.

## Discussion

This study aimed to investigate the relationship between CAR and the severity of AP, as well as its predictive value for hospital discharge timing. In settings where advanced imaging and laboratory resources may be limited, cost-effective and straightforward biomarkers like CAR are critical for timely diagnosis and triage. Our results demonstrated that CAR is significantly associated with AP severity. Notably, higher CAR values were linked to more severe disease. Patients in the highest CAR quantile (>30.5) had the greatest proportion of severe pancreatitis (45%), whereas no severe cases were observed in the lowest quantile (≤6.7). Additionally, a statistically significant positive correlation between CAR and hospital stay duration suggests its utility in predicting hospitalisation burden.

ROC analysis confirmed CAR as a reliable marker for identifying patients at risk of severe disease, showing excellent discriminatory capacity (AUC 0.862). The optimal cutoff identified by Youden’s index (18.39) yielded 100% sensitivity and 69.4% specificity, while a lower cutoff of 10.28 maintained 100% sensitivity but reduced specificity to 40.3%. Both cutoffs ensured that no severe cases were missed, underscoring CAR’s potential as a highly sensitive tool for early risk stratification.

These findings are consistent with those of Kaplan et al., who reported a 1.52-fold increase in mortality risk per unit increase in CAR and identified a threshold of 16.28 predicting poor outcomes [19]. Although only one mortality occurred in our study, limiting statistical comparison, the strong association between CAR and AP severity supports its prognostic relevance.

AP presents with a wide clinical spectrum, from mild abdominal discomfort to life-threatening multi-organ failure. After mild AP, 80% of patients recover completely, but 20% may progress, with mortality reaching 30% [20]. About half of AP-related deaths occur within the first week, primarily due to multi-organ dysfunction syndrome (MODS), which mirrors systemic complications seen in conditions like burns, sepsis, and trauma [21].

Several scoring systems have been developed to assess AP severity, but each has limitations. The Ranson criteria, although widely used, require 48 hours for full assessment and differ based on etiology (alcohol vs. gallstones), with only moderate sensitivity (73%) and specificity (77%) for mortality prediction [22]. The Bedside Index for Severity in Acute Pancreatitis (BISAP) score offers quicker assessment but has limited sensitivity [23]. Most existing models are either time-consuming or show high variability. In contrast, CAR is derived from routinely available lab tests (CRP and albumin), offering a practical, rapid, and accessible alternative to these conventional scoring systems.

Our findings align with previous reports on AP presentation. Abdominal pain was a universal symptom, followed by vomiting (80%) and fever (8.8%), consistent with Swaroop et al. [24]. The severity distribution in our study (mild 54.4%, moderate 22.5%, severe 17.5%) closely mirrors that reported by Ignatavicius et al. [25].

Strengths of our study include its prospective design, real-time data collection, and strict inclusion/exclusion criteria, which minimized confounding. CAR emerged as a simple, low-cost biomarker that can aid early triage decisions, particularly in resource-limited settings.

This study has some limitations. It was conducted in a single tertiary care center with a modest sample size, and only 14 severe cases were included, which may limit statistical power and overestimate the predictive accuracy of CAR. In addition, severity classification was unavailable for four patients, meaning the ROC analysis was restricted to 76 cases, which may have slightly reduced robustness. The study population may not represent other healthcare settings, reducing generalizability. CRP and albumin were measured only at admission, so dynamic changes over the disease course were not assessed. In addition, outcomes such as ICU admission, interventions, and mortality could not be robustly analyzed due to low event numbers. Finally, CAR was not externally validated or directly compared with established scoring systems, limiting conclusions about its relative utility. Larger multicenter studies with diverse cohorts and external validation are needed to confirm these findings. In many low- and middle-income countries, where advanced diagnostics and scoring systems may not be feasible, rapid, low-cost biomarkers are vital. CAR fulfills this role effectively. The lower cutoff (10.28) ensures high sensitivity and may be used as a screening tool to avoid missing severe cases, albeit with some over-triage. The higher cutoff (18.39) offers better specificity and may help refine patient risk categorization.

CAR shows promise as a practical tool for early identification of high-risk patients, guiding closer monitoring, early interventions, and timely referrals. Its simplicity, cost-effectiveness, and high sensitivity make it a valuable adjunct to existing scoring systems, particularly in resource-constrained environments.

## Conclusions

The CAR is a significant predictor of the severity of acute pancreatitis. Patients presenting with low CAR values have a lower risk of developing severe AP. Findings highlight that CAR, as an inexpensive, repeatable, and non-invasive marker based on systemic inflammation, serves as an independent predictor of severity in AP patients, particularly in resource-constrained settings.

## References

[1] Pannala R, Kidd M, Modlin IM (2009). Acute pancreatitis: a historical perspective. Pancreas.

[2] Gautam AK, Dewan KR, Shrestha R, Vijaya KC (2023). Clinical profile and outcome of acute pancreatitis in a tertiary health care center of Nepal. J Coll Med Sci Nepal.

[3] Bhattarai S, Gyawali M (2020). Clinical profile and outcomes in patients with acute pancreatitis attending a teaching hospital at Gandaki province, Nepal. J Coll Med Sci Nepal.

[4] Trikudanathan G, Yazici C, Evans Phillips A, Forsmark CE (2024). Diagnosis and management of acute pancreatitis. Gastroenterology.

[5] Kylänpää L, Rakonczay Z Jr, O'Reilly DA (2012). The clinical course of acute pancreatitis and the inflammatory mediators that drive it. Int J Inflam.

[6] Kant N, Beij A, Verdonk RC, van Hooft JE, Voermans RP, Spanier MB, Doggen CJ (2024). Early discharge of patients with mild acute pancreatitis - a scoping review. Pancreatology.

[7] Fagenholz PJ, Fernández-del Castillo C, Harris NS, Pelletier AJ, Camargo CA Jr (2007). Direct medical costs of acute pancreatitis hospitalizations in the United States. Pancreas.

[8] Baron TH, Morgan DE (1999). Acute necrotizing pancreatitis. N Engl J Med.

[9] Lee DW, Cho CM (2022). Predicting severity of acute pancreatitis. Medicina (Kaunas).

[10] Bradley EL 3rd (1993). A clinically based classification system for acute pancreatitis. Summary of the International Symposium on Acute Pancreatitis, Atlanta, Ga, September 11 through 13, 1992. Arch Surg.

[11] Banks PA, Bollen TL, Dervenis C (2013). Classification of acute pancreatitis--2012: revision of the Atlanta classification and definitions by international consensus. Gut.

[12] Ranson JH, Rifkind KM, Roses DF, Fink SD, Eng K, Localio SA (1974). Objective early identification of severe acute pancreatitis. Am J Gastroenterol.

[13] Balthazar EJ, Robinson DL, Megibow AJ, Ranson JH (1990). Acute pancreatitis: value of CT in establishing prognosis. Radiology.

[14] Papachristou GI, Muddana V, Yadav D, O'Connell M, Sanders MK, Slivka A, Whitcomb DC (2010). Comparison of BISAP, Ranson's, APACHE-II, and CTSI scores in predicting organ failure, complications, and mortality in acute pancreatitis. Am J Gastroenterol.

[15] Sheinenzon A, Shehadeh M, Michelis R, Shaoul E, Ronen O (2021). Serum albumin levels and inflammation. Int J Biol Macromol.

[16] Matowicka-Karna J (2016). Markers of inflammation, activation of blood platelets and coagulation disorders in inflammatory bowel diseases. Postepy Hig Med Dosw (Online).

[17] Zhou T, Zhan J, Hong S (2015). Ratio of c-reactive protein/albumin is an inflammatory prognostic score for predicting overall survival of patients with small-cell lung cancer. Sci Rep.

[18] Poudel R, Chandra K, Shah S, Mahasheth N, Mishra S, Paudel K (2019). Prevalence of acute abdomen admission in surgery ward at tertiary care center of Nepal. J Univ Coll Med Sci.

[19] Kaplan M, Ates I, Akpinar MY (2017). Predictive value of C-reactive protein/albumin ratio in acute pancreatitis. Hepatobiliary Pancreat Dis Int.

[20] Robert JH, Frossard JL, Mermillod B (2002). Early prediction of acute pancreatitis: prospective study comparing computed tomography scans, Ranson, Glascow, acute physiology and chronic health evaluation ii scores, and various serum markers. World J Surg.

[21] Wilson C, Heath DI, Imrie CW (1990). Prediction of outcome in acute pancreatitis: a comparative study of APACHE II, clinical assessment and multiple factor scoring systems. Br J Surg.

[22] Eachempati SR, Hydo LJ, Barie PS (2002). Severity scoring for prognostication in patients with severe acute pancreatitis: comparative analysis of the Ranson score and the APACHE III score. Arch Surg.

[23] Yang YX, Li L (2016). Evaluating the ability of the bedside index for severity of acute pancreatitis score to predict severe acute pancreatitis: a meta-analysis. Med Princ Pract.

[24] Swaroop VS, Chari ST, Clain JE (2004). Severe acute pancreatitis. JAMA.

[25] Ignatavicius P, Gulla A, Cernauskis K, Barauskas G, Dambrauskas Z (2017). How severe is moderately severe acute pancreatitis? Clinical validation of revised 2012 Atlanta Classification. World J Gastroenterol.

